# Closing the cancer care gap: What does it mean for the Americas?

**DOI:** 10.1016/j.lana.2022.100208

**Published:** 2022-02-15

**Authors:** The Lancet Regional Health– Americas

The World Cancer Day celebration on Feb 4 this year calls us to reflect on the unfair reality that, today, who you are and where you live can define if you'll survive or die from cancer. In the region of the Americas, there are huge disparities in cancer deaths. The 1.4 million reported cancer-related fatalities in 2020 were evenly split between northern America and Latin America and the Caribbean; however, the number of cases is 1·74-times higher for the former region (2 556 862 *vs* 1 470 274), exposing the brutal consequences of the inequity in access to cancer care in the region. According to the Global Cancer Observatory report from December 2020, if no actions are taken, the number of incident cancer cases is expected to increase by 54·7% in the Americas by 2040, with countries with low and medium Human Development Index (HDI) bearing the greatest relative increases: 70% for Central America and 68% for Brazil. As distressing as these numbers might be, much can be done to avoid such predictions from becoming a reality. This year's World Cancer Day theme—“*Close the care gap*”—brings attention to the fact that, despite living in a time of scientific and medical advances, with new drugs and treatments extending survival and improving the life quality of those fighting cancer and with the highest cure rate ever seen, a substantial part of the human population does not have access to quality cancer care.

Nearly half of patients living with cancer are aged 65 years or older, and yet older people are frequently excluded from clinical trials designed to evaluate the safety and effectiveness of cancer drugs. The implications of being excluded go beyond the generalisability of the trials’ findings, as in many low- and middle-income countries (LMICs), a patients’ participation in a clinical trial could be their only chance to access the cancer treatment needed. The overlapping of those receiving the poorest cancer care with a known vulnerable population comprised of racial and ethnic minorities, Indigenous peoples, the LGBTQI+ community, older adults, people living in remote areas, and those from low socioeconomic backgrounds is not surprising. From an action point, identifying and acknowledging the key barriers to prevention, diagnosis, and treatment for these populations is essential to end inequity in cancer care. A network analysis by Fonseca and colleagues published in *The Lancet Regional Health Americas* showed that more than half of Brazilian patients (49·2% to 60·7%) needed to travel beyond their municipality of residence for cancer treatment, and those living in the northern and midwestern regions (encompassing both the lower HDI and most of the Indigenous areas) suffered the greatest impact, with a reduced number of specialised hospitals in the area and the greatest distances to the main cancer care hubs. In another publication, Bretas and colleagues discussed the barriers to advance breast cancer care in the country and identified the delayed start of treatment post-diagnosis as a limiting issue, mainly associated with poorly coordinated services.

To face and fight a problem, it is necessary to identify it, understand its roots and causes, and define targeted, specific strategies to address it. Imperative to designing a strategic action plan is the availability of reliable, updated, and comprehensive data. Currently, high-quality cancer registries are available for fewer than 3% and 10% of Central and South America's populations, respectively. Indigenous peoples account for a great share of this population, close to being the majority in several countries, such as Bolivia and Guatemala; however, the scarce and poor-quality data related to cancer make this population statistically invisible and, as a consequence, their particular challenges to access cancer care—including geographical isolation, language barrier, and fear of discrimination—are often not accounted for when public health policies are developed. Unsurprisingly, cancer mortality rates in Indigenous people tend to be much higher than in non-Indigenous people, greatly due to late-stage diagnosis, lack of preventive screening and informative programmes, and reduced access to primary health care.

The Integrated Cancer Control Initiative in Latin America (ICCI-LA) is a policy study on health systems response to cancer control in four countries in Latin America—Argentina, Brazil, Chile, and Colombia—with a final objective to generate recommendations that will help guide the development of a National Cancer Control Plan (NCCP). In their most recent publications, among their highest priority and high-priority recommendations, all four countries listed “strengthen national population-based cancer registries”. Although all four groups identified the fragmentation within the health system as a key challenge responsible for inequitable health outcomes, costs, and quality of services among geographical regions, none of the reports acknowledged the specific needs of marginalised populations beyond geographical accessibility. For example, adherence to preventive care is markedly lower in transgender people than in the rest of the population, partly due to discrimination from health-care practitioners. The implementation of tailored cancer screening and prevention initiatives centred on relevant anatomy and high-risk behaviours specific for transgender men and women would help increase adherence to preventive care, while equipping health-care professionals with the relevant skills and knowledge is paramount to overcome discrimination. The development of an NCCP, as proposed by the Union for International Cancer Control, can be a key strategy to fight cancer and narrow the care gap; however, its effectiveness can be jeopardised if it fails to include the unique challenges faced by vulnerable populations that would benefit the most from more inclusive, humanised approaches and culturally safe prevention initiatives. Thus, to achieve equity in cancer care any proposed national plan must act to overcome the socially imposed barriers and limitations preventing vulnerable populations from accessing adequate care.

